# Dental imaging using an ultra-high resolution photon-counting CT system

**DOI:** 10.1038/s41598-022-11281-x

**Published:** 2022-05-03

**Authors:** Maurice Ruetters, Sinan Sen, Holger Gehrig, Thomas Bruckner, Ti-Sun Kim, Christopher J. Lux, Heinz-Peter Schlemmer, Sarah Heinze, Joscha Maier, Marc Kachelrieß, Stefan Sawall

**Affiliations:** 1 Section of Periodontology, Department of Operative Dentistry, University Hospital Heidelberg, Im Neuenheimer Feld 400, 69120 Heidelberg, Germany; 2 Department of Orthodontics, University Hospital of Schleswig-Holstein, Arnold -Heller-Straße 3, 24105 Kiel, Germany; 3 Institute of Medical Biometry, University Hospital Heidelberg, Im Neuenheimer Feld 130.3, 69120 Heidelberg, Germany; 4 Department of Orthodontics, University Hospital Heidelberg, Im Neuenheimer Feld 400, 69120 Heidelberg, Germany; 5 German Cancer Research Center (DKFZ), Im Neuenheimer Feld 280, 69120 Heidelberg, Germany; 6 Institute of Forensic and Traffic Medicine, University Hospital Heidelberg, Im Neuenheimer Feld 400, 69120 Heidelberg, Germany; 7 Division of X-Ray Imaging and CT, German Cancer Research Center (DKFZ), Im Neuenheimer Feld 280, 69120 Heidelberg, Germany; 8 Medical Faculty, Ruprecht-Karls-University Heidelberg, Im Neuenheimer Feld 672, 69120 Heidelberg, Germany

**Keywords:** Dental diseases, Radiography

## Abstract

Clinical photon-counting CT (PCCT) offers a spatial resolution of about 200 µm and might allow for acquisitions close to conventional dental CBCTs. In this study, the capabilities of this new system in comparison to dental CBCTs shall be evaluated. All 8 apical osteolysis identified in CBCT were identified by both readers in all three PCCT scan protocols. Mean visibility scores showed statistical significant differences for root canals(p = 0.0001), periodontal space(p = 0.0090), cortical(p = 0.0003) and spongious bone(p = 0.0293) in favor of high and medium dose PCCT acquisitions. Overall, both devices showed excellent image quality of all structures assessed. Interrater-agreement showed high values for all protocols in all structures. Bland–Altman plots revealed a high concordance of both modalities with the reference measurements. In vitro, ultra-high resolution PCCT can reliably identify different diagnostic entities and structures relevant for dental diagnostics similar to conventional dental CBCT with similar radiation dose. Acquisitions of five cadaveric heads were performed in an experimental CT-system containing an ultra-high resolution PC detector (0.25 mm pixel size in isocenter) as well as in a dental CBCT scanner. Acquisitions were performed using dose levels of 8.5 mGy, 38.0 mGy and 66.5 mGy (CTDI16cm) in case of PCCT and of 8.94 mGy (CTDI16cm) in case of CBCT. The quality of delineation of hard tissues, root-canals, periodontal-space as well as apical osteolysis was assessed by two readers. Mean visibility scores and interrater-agreement (overall agreement (%)) were calculated. Vertical bone loss (bl) and thickness (bt) of the buccal bone lamina of 15 lower incisors were measured and compared to reference measurements by ore microscopy and clinical probing.

## Introduction

High-quality volumetric imaging of dental structures usually requires a spatial resolution that cannot be achieved by conventional clinical computed tomography (CT) systems but is available in dedicated cone-beam CT systems (CBCT)^1^. Clinical photon-counting CTs (PCCT) offer a spatial resolution of up to 200 µm and might allow for acquisitions close to conventional dental CBCTs (usually around 125 µm and up to 80 µm) with the added benefits of increased soft tissue contrast and high scan speeds^2–5^. In photon-counting detectors, the scintillator found in conventional energy-integrating clinical detectors is replaced by a semiconductor. The absorption of an incoming X-ray photon results in the formation of a charge cloud that is transported to electrodes, i.e. the pixels^6–8^. To account for the high flux rate in clinical CT, the electrodes are smaller compared to the pixels of conventional clinical CT systems allowing for a higher spatial resolution.

In dentistry, CBCT is the reference standard method for imaging dense anatomical structures of interest in three dimensions, e.g. teeth and bones^9–17^. Because of its accurate three-dimensional imaging, CBCT can be used as an additional diagnostic tool for focus searches prior to, for example, radiation therapies or regular bisphosphonate administration identifying any diseases of the teeth and surrounding tissues, thus improving potential prognostic statements and avoiding severe adverse events of dental origin, e.g. dentogenic abscesses, during the mentioned therapies^18–20^.

While CBCT is mainly used for dental or otolaryngic indications, the range of indications for CT scans in general clinical medicine is vast and manifold. Depending on a specific diagnostic question, jaws and paranasal sinuses are also imaged within these acquisitions^21^. If such CT images acquired using an ultra-high resolution photon-counting CT system could depict dental structures and disease correlates accurately enough, they could, for example, avoid required additional three-dimensional dental CBCT acquisitions for the indications mentioned before and thus reduce the radiation exposure to the patient and the psychological burden of multiple examinations in usually distinct departments. Given an ever-advancing digitalization of the documentation of the health care system, it would certainly be possible to transmit these clinical CT images to other disciplines like dentistry^22^. Incidental findings in the acquired ultra-high resolution photon-counting CT data could prevent potential dental emergencies such as dentogenic abscesses through earlier diagnosis and intervention.

Hence, hypothesis of this study is that an experimental photon-counting CT system is at least as capable as a state-of-the-art dental CBCTs for imaging tooth structures and corresponding pathologies such as apical osteolysis.

## Results

### Subjective analysis

**Figure 1:**
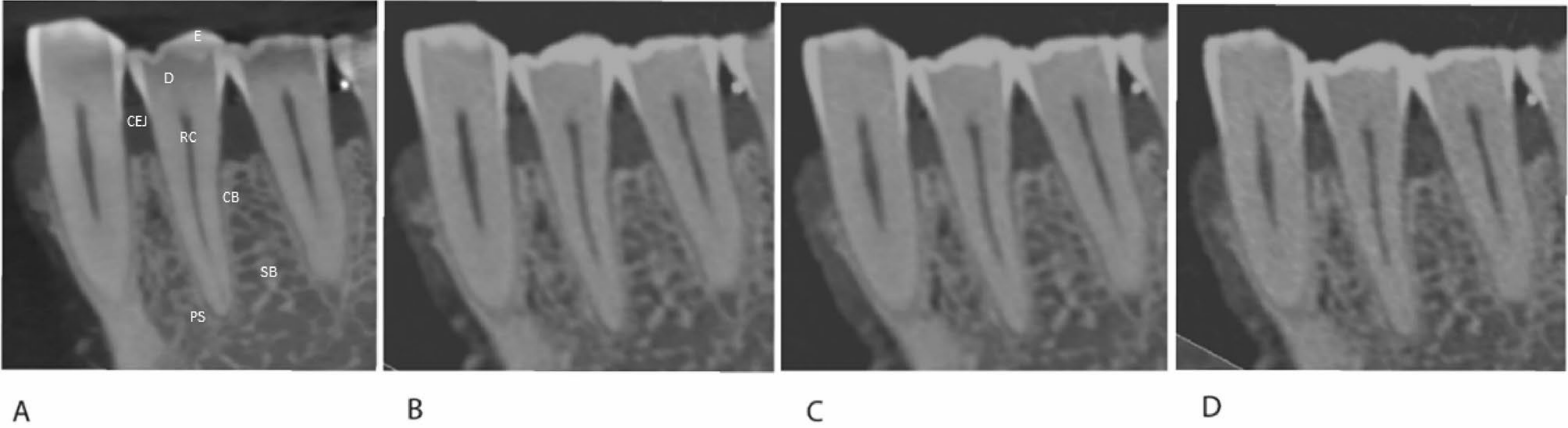
Sagittal reformations of the same region: Teeth 33, 34 and 35 in (**A**) CBCT (**B**) PCCT HD (**C**) PCCT MD (**D**) PCCT LD; *D* dentine, *E* Enamel, *CEJ* Cemento-Enamel-Junction, *RC* root canal, *PS* periodontal space, *CB* cortical bone, *SB* spongious bone. (C = 1535 HU, W = 8504 HU).

In total, 47 teeth were subjectively analyzed in CBCT, PCCT HD, PCCT MD and PCCT LD (Fig. 1). The distribution of tooth type was as follows: 21 lower front teeth, 4 upper front teeth, 13 lower molars/premolars and 9 upper molars/premolars. Due to missing enamel and presence of crowns, only 33 of 47 teeth were included for the rating of enamel and CEJ, 38 for the rating of dentine.

**Figure 2:**
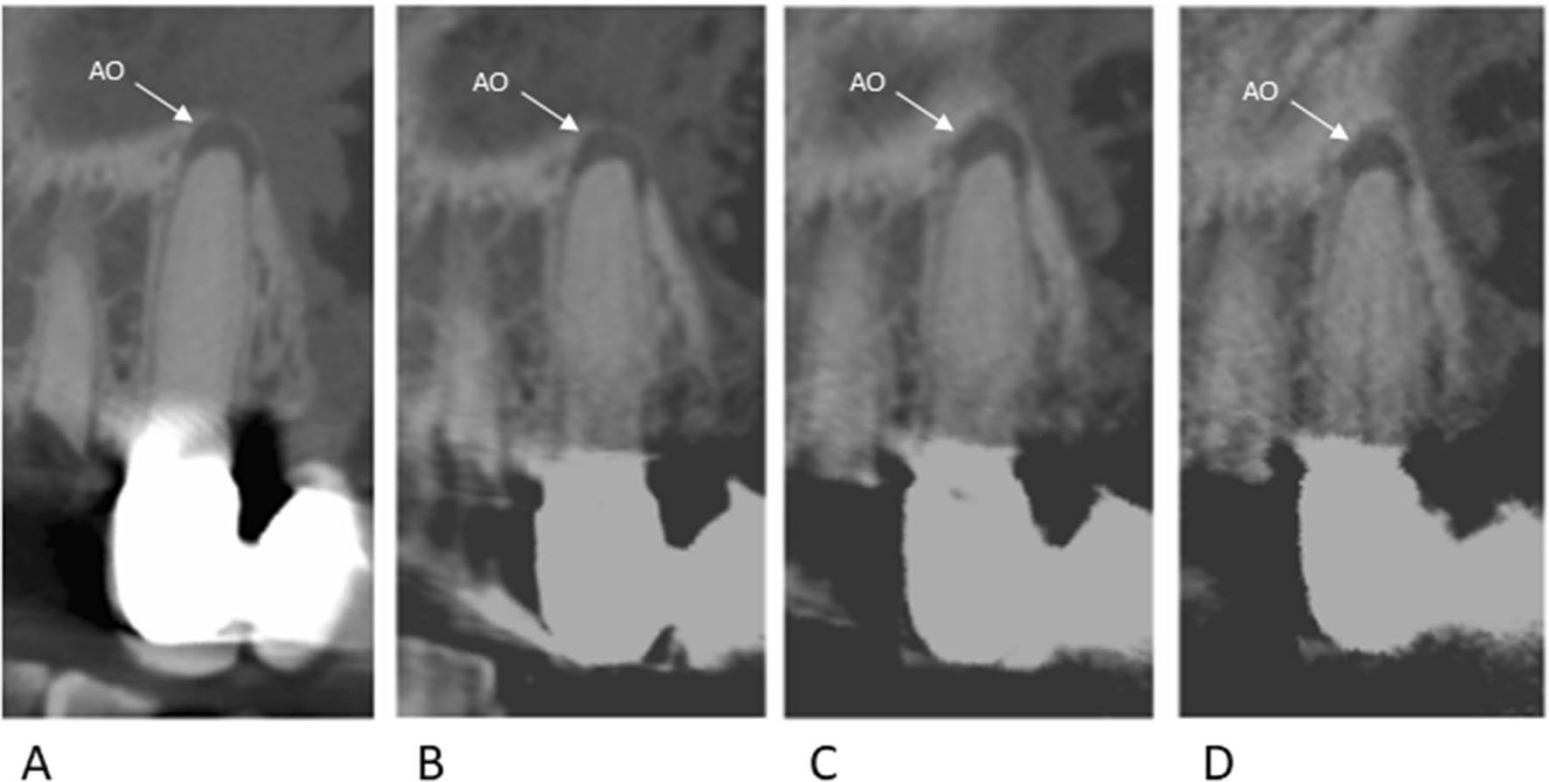
Example of an apical osteolysis of tooth 24 in all scan protocols; *AO* apical osteolysis. (C = 1535 HU, W = 8504 HU).

All 8 apical osteolysis as well as all 88 root-canals were identified in all modalities by both readers resulting in overall interrater agreement of 100% in all protocols and an overall intermodality agreement of 100% (Fig. 2).

Considering visibility scores without dichotomization, interrater agreement was high for all structures an all modalities. A total overall agreement of 100% was calculated for enamel, dentine and CEJ in all protocols. All other overall interrater agreements are shown in Table 1.

**Table 1:** Overall interrater agreements of visibility scores.

Structure	Overall agreement (%)			
	CBCT	PCCT HD	PCCT MD	PCCT LD
Enamel	100	100	100	100
Dentine	100	100	100	100
CEJ	100	100	100	100
Root canal	83	91	87	75
Periodontal space	83	100	100	91
Cortical bone	85	100	100	89
Spongious bone	98	100	100	91

After dichotomization of visibility scores to two groups (1–3 and 4–5), overall interrater agreement was 100% for the assessment of all structures in all protocols.

**Figure 3:**
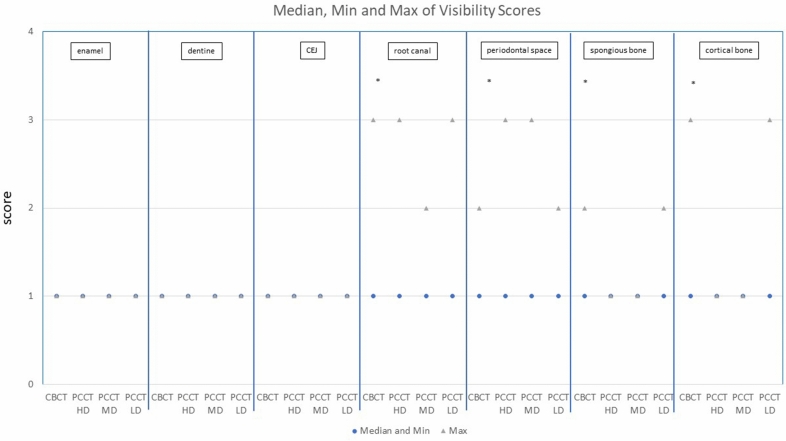
Medians and maximums of visibility scores. The asterisk (*) marks significant differences (p < 0.05).

Medians of visibility scores for all anatomic structures dependent on the protocols are shown in Fig. 3. Differences were only found for root canal, periodontal space, cortical bone and spongious bone. Overall Friedman Test showed significant differences for all parameters. Pairwise Friedman tests were used to identify the protocols resulting in these differences (Table 2). Percentage of different visibility scores assessed for each structure are shown in Table 3.

**Table 2:** Friedman Test and corresponding p-values.

Parameter	Overall p- value	CBCT vs PCCT HD	CBCT vs PCCT LD	CBCT vs PCCT MD	PCCT HD vs PCCT LD	PCCT HD vs PCCT MD	PCCT LD vs PCCT MD
Periodontal space	0.0090*	0.0143*	0.4795	0.0143*	0.0455*	1.0	0.0455*
Corticalis	0.0003*	0.0027*	0.1573	0.0027*	0.0253*	1.0	0.0253*
Spongiosa	0.0293*	0.0833	0.5637	0.0833	0.0455*	1.0	0.0455*
Root canals	0.0001*	0.0126*	0.5637	0.0126*	0.0023*	1.0	0.0003*

The asterisk (*) marks significant differences (p < 0.05).

**Table 3:** Amount of different visibility scores (%) for each structure assessed.

Tissue	Protocol	Amount of different visibility scores (%)				
		1	2	3	4	5
Enamel	CBCT	100	0	0	0	0
	PCCT HD	100	0	0	0	0
	PCCT MD	100	0	0	0	0
	PCCT LD	100	0	0	0	0
Dentine	CBCT	100	0	0	0	0
	PCCT HD	100	0	0	0	0
	PCCT MD	100	0	0	0	0
	PCCT LD	100	0	0	0	0
CEJ	CBCT	100	0	0	0	0
	PCCT HD	100	0	0	0	0
	PCCT MD	100	0	0	0	0
	PCCT LD	100	0	0	0	0
Root canal	CBCT	72	15	13	0	0
	PCCT HD	87	9	4	0	0
	PCCT MD	87	13	0	0	0
	PCCT LD	72	21	7	0	0
Periodontal space	CBCT	87	13	0	0	0
	PCCT HD	81	17	2	0	0
	PCCT MD	72	15	13	0	0
	PCCT LD	94	6	0	0	0
Spongious bone	CBCT	94	6	0	0	0
	PCCT HD	100	0	0	0	0
	PCCT MD	100	0	0	0	0
	PCCT LD	91	9	0	0	0
Cortical bone	CBCT	81	17	2	0	0
	PCCT HD	100	0	0	0	0
	PCCT MD	100	0	0	0	0
	PCCT LD	89	9	2	0	0

### Quantitative analysis

In total, 15 default sites of 15 lower human incisors were investigated radiographically and compared to clinical measurements.

The means and corresponding standard deviations of *bl* and *bt* are shown in Table 4. Turkey grouping revealed no statistical differences between any of the protocols in respect to *bl* and *bt*.

**Table 4:** Means of *bl* and *bt*.

Protocol	Variable	N	Mean [mm]	SD [mm]	Minimum [mm]	Maximum [mm]
CBCT	*bl* *bt*	15 15	**7.1** **0.4**	1.6 0.1	5.3 0.3	9.9 0.7
PCCT HD	*bl* *bt*	15 15	**7.1** **0.5**	1.6 0.1	5.2 0.3	10.0 0.7
PCCT LD	*bl* *bt*	15 15	**7.1** **0.5**	1.7 0.1	5.2 0.3	10.2 0.7
PCCT MD	*bl* *bt*	15 15	**7.1** **0.4**	1.6 0.1	5.1 0.2	10.0 0.7

*N* number of measurements, *SD* standard deviation.

**Figure 4:**
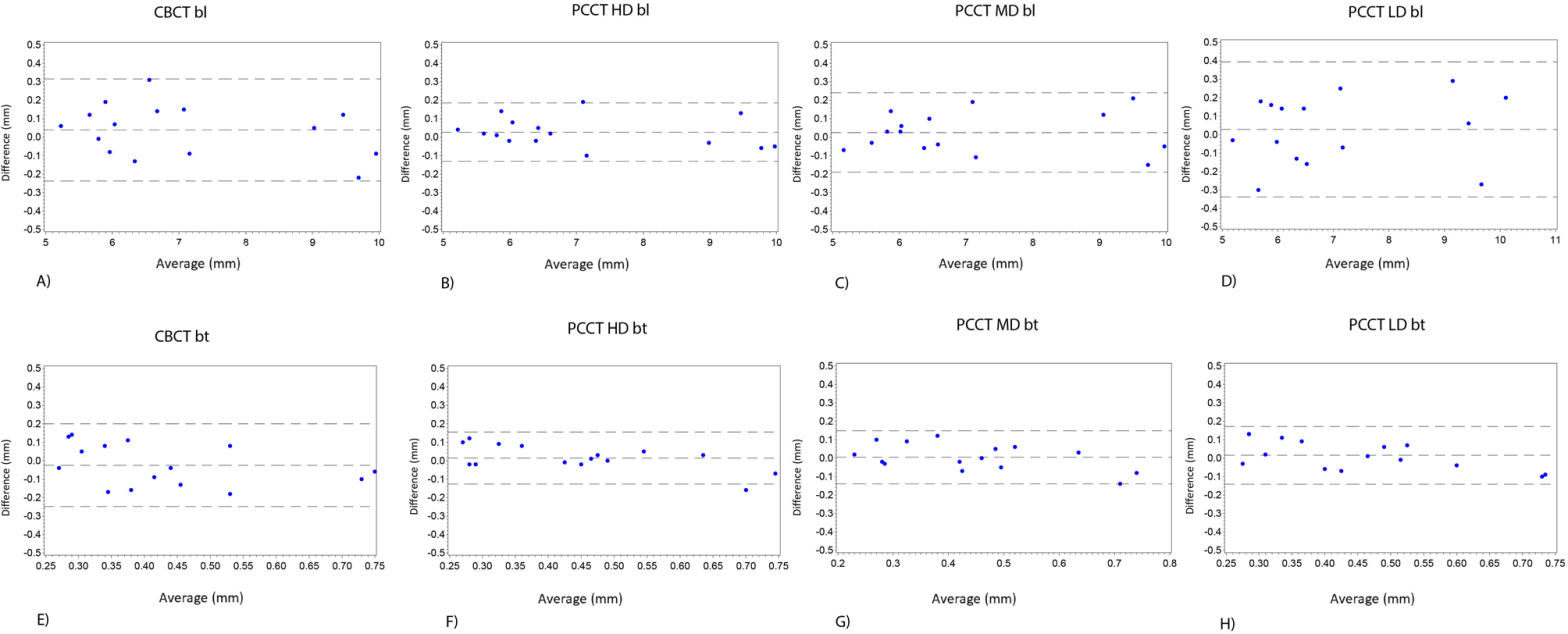
Bland–Altman-Plots: (**A–D**) Bland–Altman-Plots of *bl*-measurements of the different protocols (**E**–**H**) Bland–Altman-Plots of *bt*-measurements of the different protocols.

Means of differences of *bl* and measurements compared to the reference are shown in Fig. 4. The mean of differences of *bl*-measurements to clinical measurements were as follows: CBCT 0.039 mm (95%-limits of agreement: − 0.237 mm; 0.315 mm), PCCT HD 0.026 mm (− 0.131 mm; 0.184 mm), PCCT MD 0.024 mm (− 0.190 mm; 0.239 mm) and PCCT LD 0.028 mm (− 0.338 mm; 0.394 mm).

The mean difference of all *bt*-measurements to clinical measurements were as follows: CBCT **− **0.025 mm (95%-limits of agreement **− **0.249 mm; 0.198 mm), PCCT HD 0.014 mm (**− **0.126 mm; 0.154 mm), PCCT MD 0.004 mm (**− **0.139 mm; 0.147 mm) and PCCT LD 0.014 mm (**− **0.141 mm; 0.170 mm).

## Discussion

The results of the present study confirm that PCCT can identify and visualize the selected dental structures and disease states, at least as good as a state-of-the-art CBCT is able to.

In case of the subjective analysis, this is proven by high interrater and intermodality agreements. Occurring significant differences all favoured PCCT over CBCT. Interestingly, no significant differences were found between PCCT HD and PCCT MD as well as between CBCT and PCCT LD with respect to visibility score. This is mainly due to the noise reduction since image contrast and spatial resolution was constant for all PCCT protocols. From a clinical point of view, it should be noted that all examined structures could be identified with a clinically sufficient image quality regardless of the used protocol.

Measurements within the scope of the quantitative analysis also showed high intermodality agreements without significant differences between means of *bl* and *bt* with respect to the modality and protocol used. As can be seen from the Bland Altman Plots, all protocols showed high concordances with clinical and ore microscopy measurements without systematic over- or underestimation of vertical bone loss and bone thickness. CBCT was not superior to any of the PCCT protocols and PCCT HD was not noticeable superior to PCCT MD or PCCT LD in that matter. Within the PCCT protocols, the 95%-limits-of-agreement increased with decreasing radiation dose. This was expected since, as seen in the visibility scores, the assessment of cortical bone in the PCCT LD was significantly more ambiguous than in the protocols with higher radiation doses. The differences of *bl* were all below 1 mm and therefore within the range of measurement uncertainties of clinical measurements with periodontal probes^23,24^. For CBCT, the results are consistent with studies already performed on porcine pines and human cadavers^25,26^.

The results of the entire study may be of interest in the future for extended interpretation of data sets acquired using PCCT. The radiation dose administered in the protocol PCCT MD was intentionally chosen as 38 mGy (CTDI_16cm_) since it is well within the range of established clinical protocols for head/neck examinations^27,28^. This protocol showed excellent results with respect to the subjective and quantitative measurements. This is promising since PCCT is expected to be ubiquitously available in clinical practice in the next decades. Considering the increasing digitization in the documentation of patient data, such images could provide a significant added value for patients and treating dentists^18,20,21,29^. While not investigated herein, PCCT also offers multi-energy information due to the simultaneous acquisition of several energy bins. Such information is not available in conventional dental CBCT systems. It might be used to discriminate different materials or to provide virtual monochromatic images. However, this is a topic of future research.

### Limitations

The number of cases used for the subjective analysis with 47 teeth is acceptable. The number of sites analysed quantitatively is rather low with only 15 supplements and represents a disadvantage of this study. The case number was limited due to the number of available human donors and their dental status. However, the high levels of agreement between the different protocols and the comparison to the reference standard suggest a good reliability and precision of the different protocols.

The fact that this is an ex vivo study is another limitation. In clinical practice, artifacts in the images may occur due to various circumstances. Especially motion artifacts can cause blurring and a loss of spatial resolution^30^. However, this effect is probably more pronounced in CBCT since patients are usually standing upright whereas in PCCT they are in a prone position. While sophisticated methods exist that account for rigid motion during CBCT acquisitions, such methods are usually not available in commercial systems but are a topic of ongoing research^31–33^.

The radiographic evaluations presented in this study were performed by two dental experts since the main objective was to test and compare the capabilities of the protocols. However, it has already been shown—*e.g.* in the field of endodontics—that diagnostic results in CBCT findings were highly dependent on the experience of the examiners^13^. Similar effects must be assumed for the present study. The reconstructed images differ in terms of spatial resolution. In case of PCCT, a dedicated ultra-high resolution kernel was used. CBCT images were reconstructed using a sharp kernel. Usually, such reconstruction kernels selected in the user interface of the software not only define spatial resolution but also include dedicated artifact correction methods, e.g. for beam hardening or scattered radiation. Unfortunately, the internals of such methods are often not disclosed and hence the applied pre- and post-corrections in case of PCCT and CBCT remain unknown.

PCCT data were reconstructed using a conventional filtered backprojection algorithm since no iterative reconstruction algorithm is available for our experimental PCCT system. The use of iterative reconstruction methods might be a topic of future research since it promises a further reduction in image noise and administered radiation dose.

A mandatory prerequisite of the approach of opportunistic dental diagnostics presented herein is the availability of suitable reconstructions using ultra-high resolution kernels. Such reconstructions might not always be performed in clinical practice, yet, and a retrospective image reconstruction would require the storage of potentially large rawdata files. Hence to enable this approach, an adoption of clinical workflow is required.

## Conclusion

Overall, the results of this study are promising in terms of possible dental imaging with PCCT. Within its limitations, the study illustrated that PCCT is a highly accurate and reliable method to detect apical osteolysis as well as root canals, to describe different dental structures and to measure the buccal lamella and its thickness at lower incisors in vitro. Therefore, it may help in the future to avoid additional radiographic images in certain interdisciplinary contexts as described. Future studies need to confirm these results in the clinical setting.

## Methods

### Specimen

Acquisitions of five cadaveric human half-sectioned heads were performed. All experiments were approved by the local ethics committee (S-017/2020) and by the ethical review board of the medical association of Rhineland- Palatinate (Germany, 2021–15661). The mandibles of the used heads were fully covered by soft tissue at the time of radiographic investigations. The tongue, neck muscles, base of the skull, and cervical vertebrae were also still present. Before image acquisition, two depressions were made on the vestibular surface of the crowns of 15 lower front teeth by means of a round diamond burr (801L 314 016, Komet Dental, Gebr. Brasseler GmbH & Co. KG, Lemgo, Germany). Gel pads were used to imitate the other half of the head to achieve tissue-equivalent volumes and ensure the most lifelike radiation absorption^34^

### Imaging modalities

Experiments were performed in an experimental CT system (SOMATOM CounT, Siemens Healthineers, Forchheim, Germany) as well as in a dental CBCT scanner (Veraview X800, Morita, Japan). A summary of the used acquisition protocols can be found in Table 5.

**Table 5:** Scan protocols used throughout this manuscript.

	CBCT	PCCT HD	PCCT MD	PCCT LD
Tube voltage /kV	102	120	120	120
Tube current /mAs	89.5	350	200	50
Dose (CTDI_16cm_) /mGy	8.94	66.5	38.0	8.5
Voxel size /µm	125	134	134	134
Matrix size	641 × 641	2048 × 2048	2048 × 2048	2048 × 2048

*HD* high dose, *MD* medium dose, *LD* low dose, *CTDI* computed tomography dose index.

The ultra-high resolution photon-counting CT system used herein provides a pixel size of 250 µm in the center of rotation. It has been shown that data acquired using the ultra-high resolution mode of the PCCT show a significant noise and dose reduction when reconstructed to the lower spatial resolution of the energy-integrating detector^3^. Hence, one would assume that this scan mode will be the standard mode of operation in clinical practice since it offers ultra-high resolution data on demand and a significant dose reduction if only low resolution data are desired. However, it should be noted that the used PCCT system also offers other scan modes for example providing spectral acquisitions at lower resolutions and that the ultra-high resolution mode is currently limited to low pitch values since it can only be realized using a smaller focal spot.

Image acquisition using the PCCT system were conducted using a voltage of 120 kV and tube currents between 50 to 350 mAs resulting in a dose of 8.5 to 66.5 mGy (CTDI_16cm_, see Table 5). We define three dose levels. The low-dose (LD) protocol using only 8.5 mGy is similar to the CBCT acquisitions. The medium dose (MD) protocol using 38 mGy and the high dose protocol using 66 mGy represent dose levels observed in typical clinical examinations of the head and neck. These protocols reflect the fact that the ultra-high resolution acquisitions might not be performed as dedicated dental examinations and dental structures might only be considered retrospectively in case of incidental findings^35^.

Image reconstruction was performed with filtered backprojection using a ultra-high resolution kernel U70 providing a spatial resolution of 16 lp/cm (modulation transfer function at 10% modulation) onto a matrix of 2048^2^ pixels covering a field of view (FOV) of 27.5 cm and resulting in a pixel size of 134 µm. Data were reconstructed to a slice thickness of 250 µm and a slice increment of 150 µm.

Reference energy-integrating CBCT acquisitions were performed with a tube current of 5 mA and a tube voltage of 102 kV. Each acquisition was performed with a dose of 8.94 mGy (CTDI_16cm_). Image reconstruction was performed using a sharp kernel with an isotropic voxel size of 125 µm onto a matrix of 641 × 641 pixels covering a FOV of 8 × 8 cm^2^_._

### Clinical measurements

**Figure 5:**
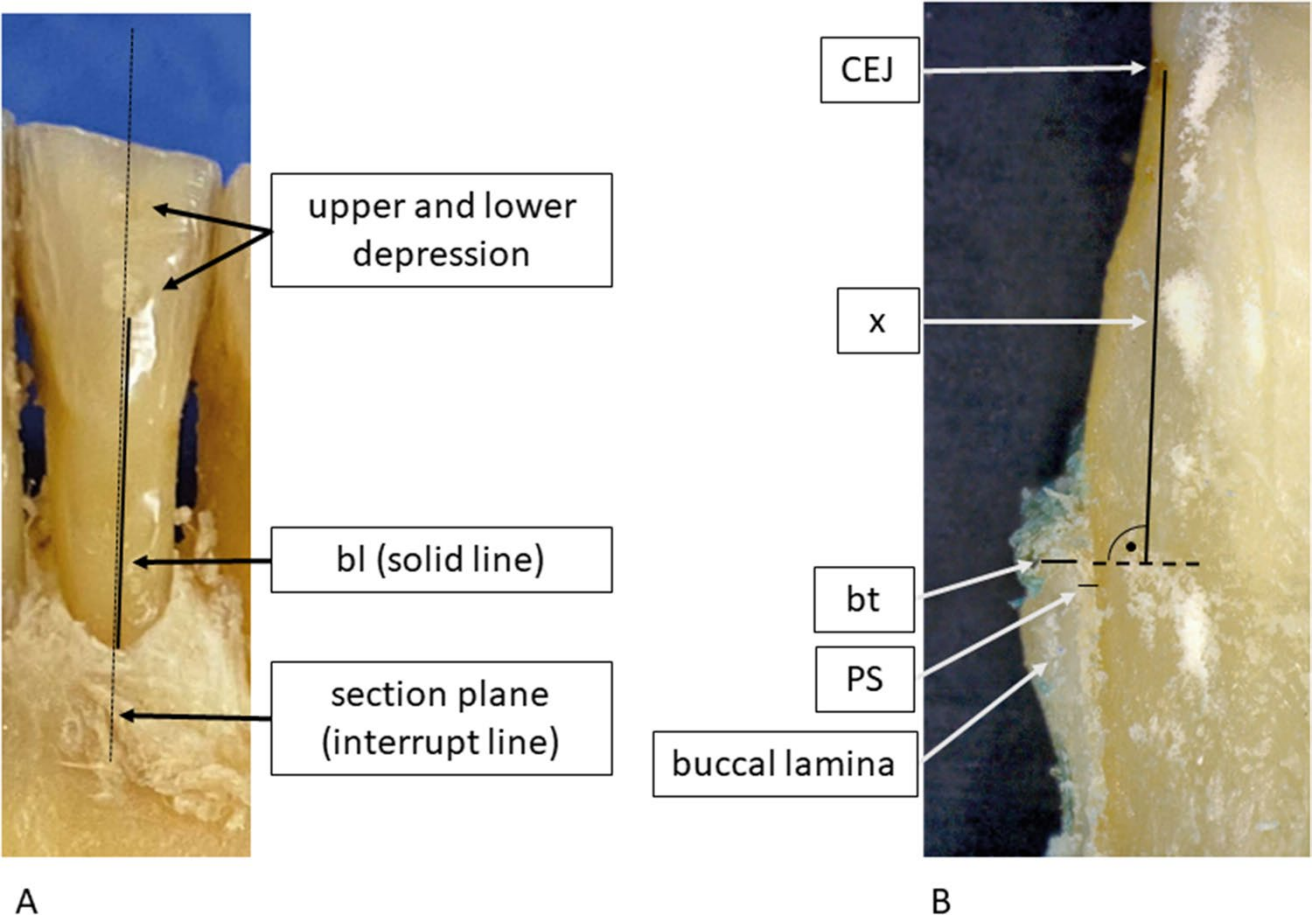
Schemes of (**A**) *bl* measurements (**B**) *bt* measurements (ore microscopy). *Bl* vertical bone loss from the most apical point of the lower depression to the most coronal point of the buccal bone, *CEJ* Cemento-Enamel-Junction, *x* random distance to a point within the first mm of the apical region of the buccal bone lamella, *bt* bone thickness, *PS* periodontal space.

Following image acquisition, the gingiva was removed. The distance of the most apical point of the lower cavity in the axis of both depressions to the most coronal point of the bone lamella was measured with a periodontal probe (FloridaProbe, FloridaProbe Dental Services UG Germany) with a 0.1 mm scale in the axis of the previously milled depressions (Fig. 5A). Thus, a reference standard for vertical bone loss (*bl*) measurements was established. These measurements were performed by one calibrated experienced examiner. For calibration, the investigator had to successfully reproduce (relative agreement of 95%) the principal investigator’s (T.K.) bone-sounding measurements of clinical attachment loss at 168 sites on a standardized *ex-vivo* reference model with a transparent gingiva (Co. M. Tech, Korea). Hereinafter, these measurements are referred to as “reference measurements”.

Sagittal sections of the teeth and their adjacent bone were prepared in the plane of the previously milled depressions. Reflected light micrographs were taken of these sagittal sections at 34 × magnification (Smartzoom 5, Carl Zeiss Microscopy, Jena, Germany). A distance *x* was determined that extended from the cemento-enamel-junction (CEJ) to a point within the first millimeters of the apical region of the buccal bone lamella. The bone thickness (*bt*) was measured at this exact location by two investigators in consensus. These measurements served as the reference standard for *bt* (Fig. 5B).

### Image review

All reconstructions were exported in DICOM format to the application RadiAnt Dicom viewer (Medxant, Poznan, Poland). Windowing and levelling were allowed. Evaluations were performed by two dentists (M.R., H.G.) blinded to each other with more than eight years (M.R.) and 15 years (H.G.) of experience. All evaluations were performed on the same monitor (RadiForce MX315W, 31.1 inch, EIZO, Mönchengladbach, Germany) in the same dark room.

### Subjective image analysis

For subjective image analysis, the quality of delineation of the following tissues and correlates of disease were used as figures of merit for comparison of both technologies and were assessed by the two experienced readers at 47 teeth using a five-point rating scale (1 = excellent, 2 = good, 3 = moderate, 4 = poor, 5 = not visible): enamel (E) (if existent), dentine (D), cemento-enamel-junction (CEJ), root-canals (RC), apical foramen (AF), periodontal space (PS), spongiosa (S) and cortical bone (CB). Moreover, the readers judged if an apical osteolysis was present and assessed the number of root-canals at each tooth. For calibration, the readers analyzed and discussed corresponding structures in 20 different sites until agreement was reached prior to image review. To enable assessment of interrater and intrarater reliability, both investigators rated twice for each protocol, with a two-week break.

### Quantitative image analysis

For quantitative image analysis, the vertical bone loss (*bl*), i.e. the distance from the most apical point of the lower cavity in the axis of both depressions to the most coronal point of the bone lamella, and the horizontal bone thickness (*bt*), i.e. the thickness of the buccal bone at a distinct site, were measured at 15 lower incisors by one calibrated investigator (M. R.). The orientation procedure was as follows: (1) The two depressions were identified, and the axis of the coronal plane was placed through the centers of the depressions. (2) The axial slice was then aligned with the lower depression. (3) In the sagittal, measurements of *bl* were taken. Afterwards, the distinct site corresponding to the distinct distance of the microscopy was identified and *bt* was measured. For calibration of the measurement protocol, the investigator performed corresponding measurements on 10 CBCT scans of different human mandibles to this study and discussed them with a second experienced investigator until agreement was reached. The investigator was blinded to the results of the corresponding measurements in all modalities as well as to the clinical measurements.

### Statistical analysis

The empirical distribution of continuous data was documented with means, standard deviations, minimum and maximum, categorical data were documented with absolute and relative frequencies. Differences between clinical and radiographic measurements were described with mean differences and corresponding standard deviations. Bland Altman plots with 95% limits of agreement were created, analyses of variance with repeated measurements including Tukey’s multiple comparisons were calculated to evaluate possible differences between groups.

Analysis of visibility scores includes calculating of overall agreement (%) by dividing the sum of identical finding with the total number of observations. Means and standard deviations of visibility scores were calculated. Overall Friedman test was calculated to examine possible differences between groups, and pairwise Friedman tests were calculated in case of significant findings to identify the protocols corresponding to these differences. Due to the nature of this study as explorative data analysis *p*-values are of descriptive nature and has no confirmatory value.

### Ethical approval

All procedures performed in studies involving human participants were in accordance with the ethical standards of the institutional and national research committee and with the 1964 Helsinki declaration and its later amendments or comparable ethical standards. The study was approved by the ethical review board of the University of Heidelberg, Germany (S-017/2020) and by the ethical review board of the medical association of Rhineland-Palatinate (Germany) (2021–15661).

### Informed consent

Both ethical review boards agreed, that no additional informed consent was required because human donors have already given their consent beforehand for donation of their body to the Department of Anatomy and Cell Biology of the University of Heidelberg for research.

## Supplementary Information

Supplementary Information.

## Data Availability

All data generated or analysed during this study are included in this published article and the supplementary file.

## References

[1] Nardi C, Talamonti C, Pallotta S (2017). Head and neck effective dose and quantitative assessment of image quality: A study to compare cone beam CT and multislice spiral CT. Dentomaxillofac. Radiol..

[2] Schlemmer, H.P. The eye of the CT scanner: The story of learning to see the invisible or from the fluorescent screen to the photon-counting detector. Rofo. 2021. 10.1055/a-1308-2693 33735934

[3] Klein L, Dorn S, Amato C (2020). Effects of detector sampling on noise reduction in clinical photon-counting whole-body computed tomography. Invest Radiol..

[4] Lell MM, Kachelriess M (2020). Recent and upcoming technological developments in computed tomography: High speed, low dose, deep learning. Multienergy. Invest Radiol..

[5] Rajendran K, Petersilka M, Henning A (2021). Full field-of-view, high-resolution, photon-counting detector CT: Technical assessment and initial patient experience. Phys. Med. Biol..

[6] Danielsson M, Persson M, Sjolin M (2021). Photon-counting x-ray detectors for CT. Phys. Med. Biol..

[7] Taguchi K, Iwanczyk JS (2013). Vision 20/20: Single photon counting x-ray detectors in medical imaging. Med Phys..

[8] Wehrse, E., Klein, L., Rotkopf, L.T., *et al.* Photon-counting detectors in computed tomography: from quantum physics to clinical practice. *Radiologe*. 2021 10.1007/s00117-021-00812-8 33598788

[9] Antony DP, Thomas T, Nivedhitha MS (2020). Two-dimensional periapical, panoramic radiography versus three-dimensional cone-beam computed tomography in the detection of periapical lesion after endodontic treatment: A systematic review. Cureus..

[10] Kang JH, Lee KS, Oh MG (2014). The incidence and configuration of the bifid mandibular canal in Koreans by using cone-beam computed tomography. Imaging Sci. Dent..

[11] Leite GM, Lana JP, de Carvalho MV (2014). Anatomic variations and lesions of the mandibular canal detected by cone beam computed tomography. Surg. Radiol. Anat..

[12] Mohanty R, Rout P, Singh V (2020). Preoperative anatomic evaluation of the relationship between inferior alveolar nerve canal and impacted mandibular third molar in a population of Bhubaneswar, Odisha, Using CBCT: A hospital-based study. J. Maxillofac. Oral Surg..

[13] Parker JM, Mol A, Rivera EM (2017). Cone-beam computed tomography uses in clinical endodontics: Observer variability in detecting periapical lesions. J. Endod..

[14] Walter C, Schmidt JC, Dula K (2016). Cone beam computed tomography (CBCT) for diagnosis and treatment planning in periodontology: A systematic review. Quintessence Int..

[15] Walter C, Schmidt JC, Rinne CA (2020). Cone beam computed tomography (CBCT) for diagnosis and treatment planning in periodontology: Systematic review update. Clin. Oral Investig..

[16] Walter C, Weiger R, Dietrich T (2012). Does three-dimensional imaging offer a financial benefit for treating maxillary molars with furcation involvement?—A pilot clinical case series. Clin. Oral Implant Res..

[17] Walter C, Weiger R, Zitzmann NU (2010). Accuracy of three-dimensional imaging in assessing maxillary molar furcation involvement. J. Clin. Periodontol..

[18] Schiegnitz, E., Al-Nawas, B., Hoefert, S,, *et al.* S3-Leitlinie Antiresorptiva-assoziierte Kiefernekrosen. AWMF Registernummer: 007–091. 2017.

[19] Schulze, R., Deppe, H., Betz, W., *et al.* s2k-Leitlinie: Dentale digitale Volumentomographie. AWMF Registernummer: 083–005. 2013.

[20] Krüger, M., Hautmann, M., Bartella, A., *et al.* S2k-Leitlinie: Infizierte Osteoradionekrose (IORN) der Kiefer, AWMF-Registernummer: 007/046. 2018.

[21] Flohé, S., Ruchholtz, S., Eikermann, M., *et al.* Deutsche Gesellschaft für Unfallchirurgie e.V. (DGU): S3 – Leitlinie Polytrauma / Schwerverletzten-Behandlung, AWMF Register-Nr. 012/019. 2016.

[22] Rieke N, Hancox J, Li W (2020). The future of digital health with federated learning. NPJ Digit. Med..

[23] Osborn J, Stoltenberg J, Huso B (1990). Comparison of measurement variability using a standard and constant force periodontal probe. J. Periodontol..

[24] Shayeb KNAA, Turner W, Gillam DG (2014). Periodontal probing: A review. Primary Dent. J..

[25] Ruetters, M., Gehrig, H., Kronsteiner, D., *et al.* Ex-vivo imaging of buccal and oral periodontal bone with low-dose CBCT in porcine jaws. Dentomaxillofac. Radiol. 2021:accepted. 10.1259/dmfr.20210233 PMC8693329 34233504

[26] Ruetters, M., Gehrig, H., Kronsteiner, D., *et al.* Ex-vivo assessment of the buccal and oral bone by CBCT J. Orofac. Orthop. / Fortschritte der Kieferorthopädie. 2021:accepted. 10.1007/s00056-021-00335-w PMC9852115 34370050

[27] Atli E, Uyanik SA, Oguslu U (2021). Radiation doses from head, neck, chest and abdominal CT examinations: An institutional dose report. Diagn. Interv. Radiol..

[28] Khoramian D, Sistani S, Firouzjah RA (2019). Assessment and comparison of radiation dose and image quality in multi-detector CT scanners in non-contrast head and neck examinations. Pol. J. Radiol..

[29] Wolff K-D, Bootz F, Beck J, et al. Leitlinienprogramm Onkologie (Deutsche Krebsgesellschaft, Deutsche Krebshilfe, AWMF): S3-Leitlinie Diagnostik und Therapie des Mundhöhlenkarzinoms, Version 3.0, AWMF Registernummer: 007/100OL https://www.leitlinienprogramm-onkologie.de/leitlinien/mundhoehlenkarzinom/ 2021.

[30] Schulze R, Heil U, Gross D (2011). Artefacts in CBCT: A review. Dentomaxillofac. Radiol..

[31] Moratin J, Berger M, Ruckschloss T (2020). Head motion during cone-beam computed tomography: Analysis of frequency and influence on image quality. Imaging Sci. Dent..

[32] Santaella GM, Wenzel A, Haiter-Neto F (2020). Impact of movement and motion-artefact correction on image quality and interpretability in CBCT units with aligned and lateral-offset detectors. Dentomaxillofac. Radiol..

[33] Spin-Neto R, Matzen LH, Schropp LW (2018). An ex vivo study of automated motion artefact correction and the impact on cone beam CT image quality and interpretability. Dentomaxillofac. Radiol..

[34] McGarry CK, Grattan LJ, Ivory AM (2020). Tissue mimicking materials for imaging and therapy phantoms: A review. Phys. Med. Biol..

[35] Paulo G, Damilakis J, Tsapaki V (2020). Diagnostic Reference Levels based on clinical indications in computed tomography: A literature review. Insights Imaging..

